# CSF APOE Secondary Glycosylation Predicts Cognitive Decline in Mild Cognitive Impairment

**DOI:** 10.3390/biomedicines14102206

**Published:** 2026-09-29

**Authors:** Yang Jiao, Chao Du, Yanfei Ding, Ruixuan Zhou, Aonan Zhao, Jun Liu, Yulei Deng, for the Alzheimer’s Disease Neuroimaging Initiative

**Affiliations:** 1 Department of Neurology, Ruijin Hospital Luwan Branch, Shanghai Jiao Tong University School of Medicine, Shanghai 200025, China; 2 Department of Neurology and Institute of Neurology, Ruijin Hospital Affiliated to the Shanghai Jiaotong University School of Medicine, Shanghai 200025, China; 3 Department of Stomatology, Shanghai Eighth People’s Hospital, Xuhui Branch of Shanghai Sixth People’s Hospital Affiliated to the Shanghai Jiaotong University School of Medicine, Shanghai 200000, China

**Keywords:** mild cognitive impairment, Alzheimer’s disease, APOE, secondary glycosylation

## Abstract

**Background:** Glycosylation is an important post-translational modification of apolipoprotein E (APOE) and APOE in cerebrospinal fluid (CSF) displays an additional secondary glycosylation that is not present in plasma, suggesting a brain-specific regulatory mechanism. This study investigated whether altered APOE glycosylation is associated with early cognitive decline and progression from mild cognitive impairment (MCI) to AD and further explored its potential biological relevance. **Methods:** APOE glycosylation levels were measured in participants with MCI and healthy controls (HCs). Analyses were stratified by *APOE4* genotype and examined in relation to neuroimaging markers. The data-derived cut-off values were used to assess disease progression. Single-cell transcriptomic data from the SEA-AD cohort were analyzed to characterize glycosylation-related alterations in dementia microglia. **Results:** The total APOE glycosylation in CSF and plasma did not differ significantly between the MCI and control groups. CSF secondary APOE glycosylation was lower in participants with MCI in the unadjusted analysis. It was not significantly associated with baseline white matter hyperintensity (WMH) or hippocampal volume. In exploratory analyses using a data-derived cut-off, lower CSF secondary glycosylation was associated with faster hippocampal volume decline and shorter time to AD conversion. Donor-level pseudobulk analysis of the SEA-AD cohort showed lower ST6GAL1 expression and higher APOE, LPL, and CD9 expression in donors with dementia; MGAT1 was not significantly altered overall. **Conclusions:** CSF secondary APOE glycosylation may be associated with early pathological changes and disease progression in MCI. Together with alterations in microglial glycosylation-related pathways, these exploratory findings suggest broader glycosylation dysregulation in APOE-related neurodegeneration.

## 1. Introduction

Apolipoprotein E (APOE) is the major lipid transport protein in the central nervous system (CNS), where it maintains cholesterol homeostasis, supports synaptic integrity, and facilitates amyloid-β (Aβ) clearance [1]. Among the three common APOE isoforms, APOE ε4 is the strongest genetic risk factor for late-onset Alzheimer’s disease (AD) and is associated with increased amyloid deposition, greater tau pathology, and faster cognitive decline [2]. However, APOE-related risk is not determined by genotype alone. Its biological function is also shaped by post-translational modifications, which regulate its conformation, receptor interactions, and lipid-binding properties [3].

Among these modifications, glycosylation has attracted increasing attention because of its potential relevance to APOE function and AD-related pathology. Glycosylation may affect APOE stability, trafficking, and lipid interactions and may also influence amyloid clearance and microglial-mediated Aβ clearance [3,4,5]. Previous studies have shown that APOE glycosylation differs across isoforms, with lower glycosylation generally observed in APOE4 compared with APOE2 and APOE3 [6,7]. Notably, reductions in secondary glycosylation, which is a CNS-specific modification observed in cerebrospinal fluid (CSF) but absent in plasma, are strongly associated with disease-related changes [5,8]. Decreased secondary glycosylation correlates with accelerated cognitive decline, reduced CSF Aβ_1–42_, and increased concentrations of tau, suggesting predictive values for disease progression [4,5,8].

These observations indicate that APOE glycosylation is not merely an ancillary molecular feature but may constitute a potentially relevant regulatory mechanism associated with genetic susceptibility and protein aggregation [3,9,10]. Furthermore, the CNS-specific nature of secondary glycosylation suggests that it may capture disease-relevant processes not reflected in peripheral biomarkers [5,7,11]. Growing evidence suggests that glycosylation-related pathways may also be altered in disease-relevant brain cellular states, providing a broader biological context for APOE-related dysfunction in AD. Nevertheless, important questions remain unresolved. Prior studies have primarily examined APOE glycosylation in established AD, leaving its role in prodromal stages such as mild cognitive impairment (MCI) insufficiently characterized [7]. MCI represents a transitional stage between normal cognition and dementia, with a substantial proportion of patients progressing to AD within a few years. Identifying CNS-specific molecular biomarkers capable of predicting this conversion early is therefore critical for timely intervention [12,13]. Therefore, characterizing CSF APOE secondary glycosylation in MCI and assessing its predictive value for cognitive decline may advance the development of biomarkers and inform therapeutic strategies in AD.

In this study, we conducted a longitudinal analysis of APOE glycosylation in individuals with MCI to explore its potential relevance to AD conversion. By combining molecular measurements with neuroimaging and clinical outcomes, we sought to determine whether CSF secondary APOE glycosylation could serve as an early indicator of neurodegeneration and cognitive decline. This work may provide preliminary evidence for post-translational modification as a candidate biomarker and generate hypotheses concerning APOE-related mechanisms in AD.

## 2. Methods

### 2.1. Study Participants

All data were sourced from the ADNI database (http://adni.loni.usc.edu/) (accessed on 26 January 2024). Participants were selected based on the availability of both blood and CSF primary and secondary glycosylation measurements, imaging data, and complete basic demographic information. This yielded 75 healthy control (HC) and 56 MCI participants, all of whom were included in every downstream analysis (glycosylation, imaging, and survival), with no further exclusions. The diagnosis of MCI was based on the Mayo Clinic diagnostic criteria [12,13]. All participants underwent cognitive and functional assessments, including the Mini–Mental State Examination (MMSE), Montreal Cognitive Assessment (MoCA), and Functional Activities Questionnaire (FAQ). Additionally, CSF levels of Aβ_1–42_, p-tau, t-tau, and *APOE* genotype and *APOE4* carrier status were analyzed in all the individuals. Moreover, white matter hyperintensities and hippocampal volume were also measured to predict the risk of AD in individuals with normal cognition or MCI.

### 2.2. Measurement of APOE Glycosylation Concentrations

Our study obtained the APOE glycosylation measurement data of plasma and CSF from the publicly available existing glycosylation data of ADNI. CSF and plasma samples were collected from each participant during the same study visit. APOE glycosylation and secondary glycosylation were quantified using a mass spectrometric immunoassay (MSIA) approach. APOE was captured from plasma and CSF samples with antibody-derivatized MSIA tips and analyzed by MALDI-TOF mass spectrometry (Bruker Autoflex III, Bremen, Germany). Mass spectra were calibrated, baseline-corrected, and smoothed, and peak intensities corresponding to APOE isoforms and glycoforms were extracted. For each isoform, total glycosylation was calculated as the summed abundance of all glycoforms relative to total APOE, while secondary glycosylation was defined as the relative abundance of secondary glycoforms within each isoform. Each plasma and CSF sample was analyzed once. The 10,000 laser shots acquired and summed for each mass spectrum represented a single analytical measurement rather than independent replicates. As repeated measurements of shared quality-control samples were not performed within or across batches, intra-assay and inter-assay coefficients of variation could not be calculated [10].

### 2.3. Assessment of CSF Aβ_1–42_, T-Tau, and P-Tau Concentrations

Briefly, Aβ_1–42,_ T-Tau, and P-Tau data were obtained from the Data Archive (https://ida.loni.usc.edu) (accessed on 26 January 2024), measured by the ADNI Biomarker Core at the University of Pennsylvania using Elecsys immunoassays on Cobas 601 analyzers (Roche Diagnostics GmbH, Mannheim, Germany), and provided as the UPENNBIOMK9.csv file. Participants were further stratified into HC and MCI subgroups according to their *APOE4* carrier status, classified as *APOE4*-positive and *APOE4*-negative [14].

### 2.4. Assessment of Hippocampal Volume

Hippocampal volumes were derived from 3T MPRAGE MRI scans processed with FreeSurfer v5.1 by the ADNI imaging core (UCSF) and obtained through the ADNI database (http://adni.loni.usc.edu/) (accessed on 26 January 2024). For longitudinal analysis, a FreeSurfer-based pipeline generated subject-specific median templates through robust image registration, enabling unbiased reconstruction, spatial normalization, and parcellation across time points. Standard preprocessing included intensity normalization, skull stripping, affine registration to Talairach space, and the segmentation of subcortical structures, followed by surface reconstruction and automated parcellation. Full details of the ADNI longitudinal imaging pipeline are available online and in prior publications [15].

### 2.5. White Matter Hyperintensity Measurement

All scans were pre-processed through a standardized pipeline. T1, T2, PD, and FLAIR were rigidly coregistered using cross-correlation as a similarity measure and previously presented optimization methods. Nonbrain tissues were manually separated from the brain on all scans. The skull-stripped T1-weighted image was then nonlinearly aligned to a minimum deformation template (MDT) based on moving control points in a multi-scale grid and using cubic spline interpolation to move image pixels between the control points. The warp is constrained such that no region is permitted to collapse entirely. The T1, T2, PD, FLAIR, and map of ground-truth FLAIR-based white matter hyperintensity (WMH) pixels were then warped to the space of the MDT image using the nonlinear alignment. This method utilizes a Bayesian Markov-Random Field (MRF) approach to WMH detection, established from a vector of three image intensities—PD, T1, and T2—associated with each image pixel. To determine a binary label for each image voxel that denotes either the presence or absence of a WMH at that voxel, the three image intensity vectors that maximize the posterior probability of the labels given the image intensity data are utilized. Comprehensive explanations of WMH measurement on the ADNI data can be accessed online at http://adni.loni.usc.edu/ (accessed on 26 January 2024) and in prior publications.

### 2.6. Data Processing and Differential Expression Analysis

Single-cell transcriptomic data (240,651 cells/nuclei) and matched donor metadata were obtained from the SEA-AD cohort. To avoid treating cells from the same donor as independent replicates, raw UMI counts were summed by gene within each donor, yielding 84 pseudobulk samples (Dementia, N = 42; No dementia, N = 42). Genes with CPM ≥ 1 in at least 10 donors were retained, and differential expression was modeled using limma-voom with Benjamini–Hochberg correction. Secondary pseudobulk analyses were stratified by Donor ID × Supertype and Donor ID × Brain Region; strata were retained only when each contained ≥20 cells per donor and ≥5 donors per diagnosis group. Both genome-wide and gene-wise BH FDR across 17 prespecified strata were reported. Primary evidence comprised a log2 fold change, 95% CI, a raw *p-*value, BH FDR, and donor counts. Cell-level Wilcoxon results were retained only as exploratory context. MGAT1 and ST6GAL1 associations with the Braak stage and *APOE* genotype were evaluated using donor pseudobulk logCPM values.

### 2.7. Data Analysis

Statistical analysis software (Prism 10.0) and R studio (version 4.3.1) were used to process the glycosylation data. Differences were assessed using Student’s *t* test for approximately normally distributed variables and the Mann–Whitney U test for non-normally distributed variables. One-way ANOVA followed by Tukey’s post hoc test was used to compare among multiple groups. Correlation analysis was performed to assess the relationship between APOE glycosylation and pathological changes and clinical symptoms of MCI and AD. Correlations were assessed using Pearson or Spearman coefficients depending on data normality. Regression analysis was further applied to explore the predictive value of APOE glycosylation in the transition from MCI to AD. In regression analyses examining the associations of APOE glycosylation with WMH volume, hippocampal volume, or time to conversion, adjustments were made for age, sex, and education level. To correct for multiple comparisons, we controlled the false discovery rate (FDR) using the Benjamini–Hochberg procedure within each prespecified family of related tests. Three families were defined: Family 1, CSF glycosylation group comparisons; Family 2, correlations with imaging markers (WMH and hippocampal volume); and Family 3, conversion analyses. The adjusted q values are reported in Supplementary Table S1. For each participant, WMH and hippocampal volume z-scores from the last available imaging follow-up were analyzed using separate multivariable linear regression models in the HC and MCI groups. Each model included a glycosylation group, the follow-up duration in months, age, sex, and the APOE ε4 carrier status. Group-specific unadjusted linear fits were displayed for descriptive purposes and were not interpreted as within-participant trajectories. Group differences in these annualized change rates between glycosylation subgroups (≤16.2 vs. >16.2) were compared using Mann–Whitney U tests. The area under the ROC curve (AUC) with a 95% confidence interval was calculated to assess overall discriminatory ability. The optimal cut-off was selected by maximizing the Youden index, calculated as J = sensitivity + specificity − 1. AUCs with 95% confidence intervals were calculated. Sensitivity and specificity at each selected cut-off were also reported. Because the cut-offs were derived and evaluated in the same dataset, these analyses were considered exploratory.

## 3. Results

### 3.1. Demographic and Clinical Characteristics

Baseline demographic and clinical profiles of participants from the ADNI cohort are summarized in Table 1. No significant group differences were observed in age (*p* = 0.150), gender distribution *(p* = 0.818) and education duration (*p* = 0.337). Cognitive assessments revealed statistically lower MMSE and MoCA scores in the MCI group (*p =* 0.004, *p* < 0.001, respectively), accompanied by a reduced functional capacity as measured by the FAQ (*p* < 0.001). The higher FAQ score in the MCI group (3.2 vs. 0.1) indicates greater impairment in instrumental daily functioning, although the absolute level of impairment remained relatively modest and was compatible with the clinical characterization of MCI in this cohort. This level of functional decline is consistent with MCI and well below the threshold typically associated with dementia. Continuous CSF Aβ_1–42_, total tau, and phosphorylated tau levels did not differ significantly between groups. The frequency of *APOE4* carriers was not significantly higher in the MCI group (*p* = 0.377).

### 3.2. Measurement of APOE Glycosylation Levels in CSF and Plasma

To evaluate the APOE glycosylation levels and secondary APOE glycosylation levels in the HC and MCI participants, we analyzed the total APOE glycosylation levels in CSF and plasma. Previous research indicate that APOE molecules derived from CSF possess two glycans, suggesting a secondary glycosylation process that is absent in plasma-derived APOE [8]. Based on this, we only detected the second APOE glycosylation levels in CSF. The results showed no significant differences in total APOE glycosylation between the MCI and HC groups for either CSF or plasma (Figure 1a,b, ns). However, secondary APOE glycosylation levels in the CSF were significantly reduced in the MCI group compared to HCs (Figure 1c, *p* = 0.041). To investigate potential genetic influences, participants were stratified based on *APOE4* gene status into carriers (+) and non-carriers (-).

Within the CSF and plasma, total glycosylation levels did not differ significantly across subgroups regardless of *APOE4* carrier status (Figure 1d,e, ns). Further analysis of CSF secondary glycosylation revealed that *APOE4* non-carrier HC participants had significantly higher levels compared to their *APOE4* carrier counterparts (Figure 1f, *p* < 0.0001). Among MCI participants, *APOE4* carriers also demonstrated lower secondary glycosylation levels compared to non-carriers (Figure 1f, *p* = 0.033). These findings highlight secondary glycosylation as a potential biomarker for cognitive decline and also underscore the potential role of *APOE4* in influencing glycosylation pathways.

### 3.3. Assessment of Glycosylation Correlations with WMH and Hippocampal Volume in MCI Based on APOE4 Carriers

WMH, visible as damaged areas in the brain’s white matter on MRI, serve as a crucial biomarker for assessing and predicting cognitive decline and related neurodegenerative and vascular diseases [16]. We next explored the relationship between APOE glycosylation levels and WMH volume to assess whether glycosylation could reliably indicate cognitive function in MCI participants. CSF total glycosylation levels in the MCI group were positively correlated with WMH volume, reaching statistical significance (Figure 2a, *p* = 0.039). This positive correlation persisted in *APOE4* carriers, though without statistical significance, while *APOE4* non-carriers demonstrated a significant association (Figure 2a, *p* = 0.023). Plasma total glycosylation showed a positive but non-significant correlation with WMH volume in the MCI group overall (Figure 2b, ns). Subgroup analysis revealed that *APOE4* carriers exhibited a non-significant negative correlation, whereas *APOE4* non-carriers showed a positive trend, also without statistical significance. CSF secondary glycosylation levels in MCI participants displayed a negative but non-significant correlation with WMH volume (Figure 2c, ns).

This trend was consistent in the subgroup analysis, where both *APOE4* carriers and non-carriers exhibited similar non-significant negative correlations. Hippocampal z-scores, derived from MRI measurements of hippocampal volume, are critical biomarkers for assessing memory function and predicting the progression of cognitive impairment [17]. For hippocampal volume, CSF total glycosylation in the MCI group showed a non-significant negative correlation (Figure 2d, ns). Subgroup analysis indicated that *APOE4* carriers exhibited a similar negative association. Plasma total glycosylation levels demonstrated a positive but non-significant correlation with hippocampal volume in the overall MCI group, with *APOE4* carriers showing a non-significant negative association (Figure 2e, ns). CSF secondary glycosylation also displayed a negative correlation with hippocampal volume, but the results were not statistically significant (Figure 2f, ns).

### 3.4. Evaluation of APOE Glycosylation Levels in Relation to WMH and Hippocampal Volume Based on Cut-Off Value

We further examined the relationships between APOE glycosylation levels and neuroimaging markers by categorizing MCI participants based on glycosylation cut-off values. For CSF total APOE glycosylation, the AUC was 0.492 (95% CI: 0.336–0.648). At a cut-off of ≤68, sensitivity was 44.44%, specificity was 62.07%, and the Youden index was 0.065. No correlation was observed between glycosylation levels and WMH volume in either subgroup (above or below the cut-off) (Figure 3a, ns). For plasma total APOE glycosylation, the AUC was 0.525 (95% CI: 0.368–0.682). At a cut-off of ≤10, sensitivity was 33.33%, specificity was 64.29%, and the Youden index was −0.024. Similarly, plasma APOE total glycosylation, with a cut-off value of 10, showed no significant association with WMH volume in either subgroup (Figure 3b, ns). Using CSF secondary APOE glycosylation ≤16.2 as the positive classification rule, the sensitivity was 33.33%, specificity was 75.86%, and Youden index was 0.092. The continuous marker yielded an AUC of 0.557 (95% CI: 0.404–0.711), with lower values indicating greater conversion risk. CSF secondary APOE glycosylation was also not significantly correlated with WMH volume in either subgroup with a cut-off value of 16.2 (Figure 3c, ns).

For hippocampal volume, CSF total glycosylation levels above the cut-off of 68 were significantly negatively correlated with hippocampal volume (Figure 3d, *p* = 0.012). However, no such correlation was observed in the subgroup with glycosylation levels below or equal to the cut-off. Plasma total glycosylation (cut-off of 10) and CSF secondary glycosylation (cut-off of 16.2) showed no significant correlations with hippocampal volume across all subgroups (Figure 3e,f, ns).

### 3.5. Impact of APOE Glycosylation Cut-Off Levels on AD Progression in MCI

To investigate the relationship between glycosylation levels and AD progression, we conducted subgroup analyses based on data-derived cut-off values. For CSF APOE total glycosylation, using a cut-off value of 68, no significant differences in AD conversion time were observed between participants with levels above or below the cut-off (Figure 4a, ns). Similarly, no significant differences were noted within the *APOE4*+ or *APOE4*- subgroups based on the same cut-off (Figure 4b,c, ns).

For plasma total glycosylation, using a cut-off value of 10, no significant differences in AD conversion time were found across MCI participants or within the *APOE4*+ and *APOE4*- subgroups (Figure 4d–f, ns).

In contrast, CSF APOE secondary glycosylation, with a cut-off value of 16.2, revealed significant differences. MCI participants with secondary glycosylation levels below or equal to the cut-off transitioned to AD significantly earlier compared to those with levels above the cut-off (Figure 4g, *p* = 0.042). A stratified analysis showed that within the *APOE4*+ subgroup, MCI participants with glycosylation levels below or equal to the cut-off transitioned to AD earlier than those with higher levels (Figure 4h, *p* = 0.036). However, no significant differences were observed in the *APOE4*- subgroup based on the same cut-off (Figure 4i, ns).

### 3.6. Evaluate the Predictive Value of CSF Secondary Glycosylation Levels

To examine the predictive value of CSF secondary glycosylation levels for structural brain changes, we analyzed their association with WMH progression and hippocampal atrophy in MCI participants using different cut-off values. When assessing WMH volume changes, HC participants, whose CSF secondary glycosylation levels were lower than or equal to the cut-off value, experienced a slower annual rate of WMH volume increase than those with a level higher than the cut-off value (Figure 5a, *p* = 0.036), while no such difference was observed in the MCI group (Figure 5b, ns).

In contrast, the analysis of hippocampal volume changes revealed distinct patterns based on cut-off levels. Participants with CSF secondary glycosylation levels below or equal to the cut-off experienced a faster rate of hippocampal atrophy compared to those with levels above the cut-off, which was particularly evident in MCI patients (Figure 5c,d, *p* < 0.05). These findings suggest that lower levels of CSF secondary glycosylation may indicate a greater susceptibility to hippocampal volume loss, highlighting its potential role as a marker for neurodegeneration severity in MCI.

### 3.7. Transcriptomic Rewiring of Glycosylation in Dementia Microglia and Clinicopathological Correlates

Donor-level pseudobulk analysis supported *APOE* up-regulation and *ST6GAL1* down-regulation but not uniform *MGAT1* up-regulation. Limma-voom analysis using 84 donors as the independent units showed a significant down-regulation of *ST6GAL1* in dementia (log2FC = −0.309, *p* = 1.16 × 10^−6^, BH FDR = 0.00148) and a significant up-regulation of *APOE* (log2FC = 0.398, BH FDR = 0.0319), *LPL* (log2FC = 0.851, BH FDR = 0.00738) and *CD9* (log2FC = 0.718, BH FDR = 0.0175) (Figure 6a). Stratified sensitivity analyses showed directionally consistent *APOE* increases across most micro-PVM states and brain regions, with genome-wide significance within DFC (log2FC = 0.575, BH FDR = 0.0261). *ST6GAL1* down-regulation maintained genome-wide significance within Micro-PVM_2, Micro-PVM_2_3-SEAAD, Micro-PVM_3-SEAAD, MEC, and MTG (Figure 6b–e). *MGAT1* was not significant overall (log2FC = 0.183, BH FDR = 0.324) and showed state/region heterogeneity, whereas *TREM2* did not pass confirmatory multiple-testing correction in any stratum (Figure 6f–j). These findings suggest that APOE/DAM activation was accompanied by ST6GAL1-dominant remodeling of the glycosylation-related transcriptional environment, rather than uniform *MGAT1* up-regulation or a robust *APOE4* dose effect.

Donor-level clinicopathological analysis identified an overall difference in *ST6GAL1* across Braak groups (Kruskal–Wallis BH FDR = 0.0408), whereas the corresponding *MGAT1* test and *APOE*-genotype omnibus tests were not significant (Figure 6k). Among the 42 dementia donors, *MGAT1* and *ST6GAL1* were inversely correlated (Spearman r = −0.456, BH FDR = 0.0214). *TREM2* retained significant donor-level correlations with *APOE* (r = 0.472, BH FDR = 0.0194) and *TYROBP* (r = 0.541, BH FDR = 0.00395), supporting its role within the coordinated DAM program despite the absence of an overall disease-group difference (Figure 6k). The broad positive synchronization previously inferred between *MGAT1* and core DAM markers was not confirmed after donor-level aggregation and multiple-testing correction. Subpopulation violin plots are presented only as descriptive cell-level distributions.

## 4. Discussion

This study assessed differences in APOE glycosylation levels in CSF and plasma between HCs and individuals with MCI and further examined their associations with the *APOE4* genotype, structural brain changes, and progression from MCI to AD. In addition, to provide a biological context for the clinical findings, we incorporated an independent single-cell transcriptomic analysis focused on glycosylation-related pathways in AD-relevant cellular states. Overall, our findings indicate that CSF secondary APOE glycosylation, rather than total APOE glycosylation, is more closely linked to early disease-related changes. Reduced CSF secondary glycosylation was not significantly associated with baseline WMH or hippocampal volume but was associated with a faster longitudinal decline in hippocampal volume in exploratory cut-off-based analyses.

No significant differences in total APOE glycosylation were detected in CSF or plasma between individuals with mild MCI and HC, indicating the limited sensitivity of this measure as a biomarker for early cognitive decline. By contrast, secondary APOE glycosylation in CSF was modestly lower in MCI, highlighting the CNS specificity of this modification and its potential role in the earliest pathological events. In this study, secondary APOE glycosylation refers to glycoform signals detected in CSF but not in plasma using the applied MSIA method [3,18,19]. This modification influences APOE’s conformational stability, transcellular trafficking, and receptor-binding affinity [20,21]. Accordingly, the loss of secondary glycosylation may reflect impaired APOE functional regulation within the CNS, which in turn could contribute to reduced amyloid clearance, neuroinflammation modulation, and synaptic homeostasis and potentially be associated with processes relevant to neurodegeneration [5].

Building on the overall cohort findings, the stratified analyses revealed that the CSF secondary glycosylation level of *APOE4* non-carriers was significantly higher than that of carriers. These findings suggest that *APOE4* may influence glycosylation through distinct biological mechanisms. Structurally, *APOE4* differs from *APOE3* and *APOE2* in a manner that may affect glycosylation site accessibility, protein stability, and downstream function. In this context, secondary glycosylation may be particularly important for maintaining APOE function in non-carriers, whereas in *APOE4* carriers, disease-related dysfunction may already be shaped by isoform-specific structural disadvantage [7,22,23]. These findings suggest that *APOE4* status should be taken into account when evaluating glycosylation-based biomarkers since combining all participants in one analysis may mask important subgroup differences.

Our imaging analyses further supported the pathological relevance of these glycosylation changes. Total APOE glycosylation in CSF was positively associated with WMH volume, with the relationship being stronger in *APOE4* non-carriers. This association may reflect an association between elevated total glycosylation and white matter pathology, potentially mediated by inflammatory processes or blood–brain barrier (BBB) dysfunction [24,25,26]. Previous neuroimaging and histopathological studies have established associations between white matter pathology, cerebral small vessel disease, chronic inflammation, and glial activation [27,28]. Glycosylation changes could contribute to these processes by modulating APOE’s interactions with lipid particles and inflammatory signaling pathways [20]. In contrast, CSF secondary APOE glycosylation showed a negative correlation trend with WMH volume. The loss of this modification could increase the susceptibility of white matter tracts to injury. The overall correlation between CSF secondary glycosylation and hippocampal volume was weak. Given that white matter tract disruption emerges early in the transition from MCI to AD, whereas hippocampal atrophy is a hallmark of later disease stages [29,30], these findings suggest that glycosylation abnormalities may exert their effects predominantly during early, rather than late, phases of pathology. Using a cut-off-based stratification, we observed that CSF secondary APOE glycosylation levels of ≤16.2 were not significantly associated with baseline WMH volume, and no significant difference in WMH progression was observed in the MCI group. These results raise the possibility that once secondary glycosylation declines past a certain point, compensatory mechanisms in the CNS may no longer be able to preserve normal function, allowing pathological cascades to advance [8,10,31]. In contrast, the negative association between CSF total APOE glycosylation above 68 and hippocampal volume suggests that marked elevation in total glycosylation may reflect a pathological shift rather than a protective one. Taken together, these observations point to the possibility that the functional impact of APOE glycosylation depends on a finely regulated balance between its specific forms and overall abundance [4]. Notably, imaging findings may be confounded by age-related atrophy and cerebrovascular pathology, as hippocampal volume loss and WMH burden reflect both neurodegenerative and vascular contributions. Although baseline age did not differ significantly between groups, within-group age effects cannot be excluded. In addition, WMH captures both vascular and non-vascular white matter changes, and detailed vascular risk factors were not consistently available for adjustment. Residual confounding therefore cannot be excluded, and future studies with comprehensive vascular risk profiling and longitudinal imaging are needed to clarify the specificity of these associations.

The additional transcriptomic analysis extends these clinical observations by suggesting that glycosylation dysregulation is also embedded within disease-associated alterations in the AD brain. In the SEA-AD dataset, dementia-associated states showed decreased *ST6GAL1* expression together with increased APOE/DAM markers (LPL, CD9). In donor-level analysis, *MGAT1* did not reach significance and showed state/region heterogeneity, and there was no robust APOE4 dose effect. *ST6GAL1* differed significantly across Braak groups, and within dementia donors *MGAT1* and *ST6GAL1* were inversely correlated. The previously suspected positive synchronization between *MGAT1* and *DAM* markers was not confirmed after donor-level aggregation and multiple-testing correction. These results indicate that glycosylation-related molecular remodeling is closely coupled with the activated microglial phenotype observed in neurodegeneration. The transcriptomic analysis was based on a previously generated dataset from an independent cohort and was not paired with direct measurements of CSF APOE secondary glycosylation. Accordingly, these findings do not establish a direct link between reduced CSF APOE secondary glycosylation and altered glycosylation-related processes in microglia. Instead, they provide complementary and hypothesis-generating evidence that these observations may represent related aspects of broader glycosylation dysregulation associated with neurodegeneration [32,33]. The proposed relationships should therefore be regarded as a conceptual framework rather than an established causal pathway. Future studies integrating paired CSF glycosylation and transcriptomic data from the same individuals, together with experimental validation, are needed to test this model.

Despite the potential clinical relevance, this study provides novel evidence linking CSF secondary glycosylation of APOE to AD-related pathology, yet several limitations should be noted. First, the overall sample size was limited, and APOE ε4 stratification further reduced subgroup numbers, limiting statistical power to detect small-to-moderate effects. Non-significant results should therefore not be interpreted as evidence of no between-group differences, as they may reflect small true effects or imprecise estimates. These subgroup findings remain exploratory and require validation in larger independent cohorts. We have presented the original *p*-values and the FDR-corrected *p*-values in the Supplementary File. Second, while the glycosylation assay enabled the quantification of primary and secondary glycoforms, it did not capture the full spectrum of glycan structures. Furthermore, our transcriptomic profiling was limited to microglia, while astrocyte-mediated glycosylation and phagocytic pathways, such as those involving ACKR3/CXCL12 [34], may also contribute to synaptic homeostasis and warrant future investigation. Finally, most analyses were cross-sectional, and longitudinal validation in larger multi-center cohorts will be important to establish temporal dynamics. Despite these limitations, the present study underscores the clinical relevance of APOE secondary glycosylation and highlights its potential as a promising biomarker for early neurodegenerative changes.

## 5. Conclusions

In this exploratory ADNI sample, CSF secondary APOE glycosylation was lower in participants with MCI than in cognitively healthy controls, whereas total APOE glycosylation in CSF and plasma did not differ significantly between groups. Lower CSF secondary glycosylation was associated with faster hippocampal volume loss and shorter time to AD conversion in exploratory analyses based on a data-derived cut-off. Donor-level pseudobulk analysis of the independent SEA-AD cohort identified lower ST6GAL1 expression and higher APOE, LPL, and CD9 expression in donors with dementia, but these transcriptomic findings were not directly linked to CSF APOE glycoforms. Given the small sample size, the lack of assay replication, multiple testing, and the absence of independent cut-off validation, these findings should be considered hypothesis-generating and require confirmation in larger longitudinal cohorts.

## Figures and Tables

**Figure 1:**
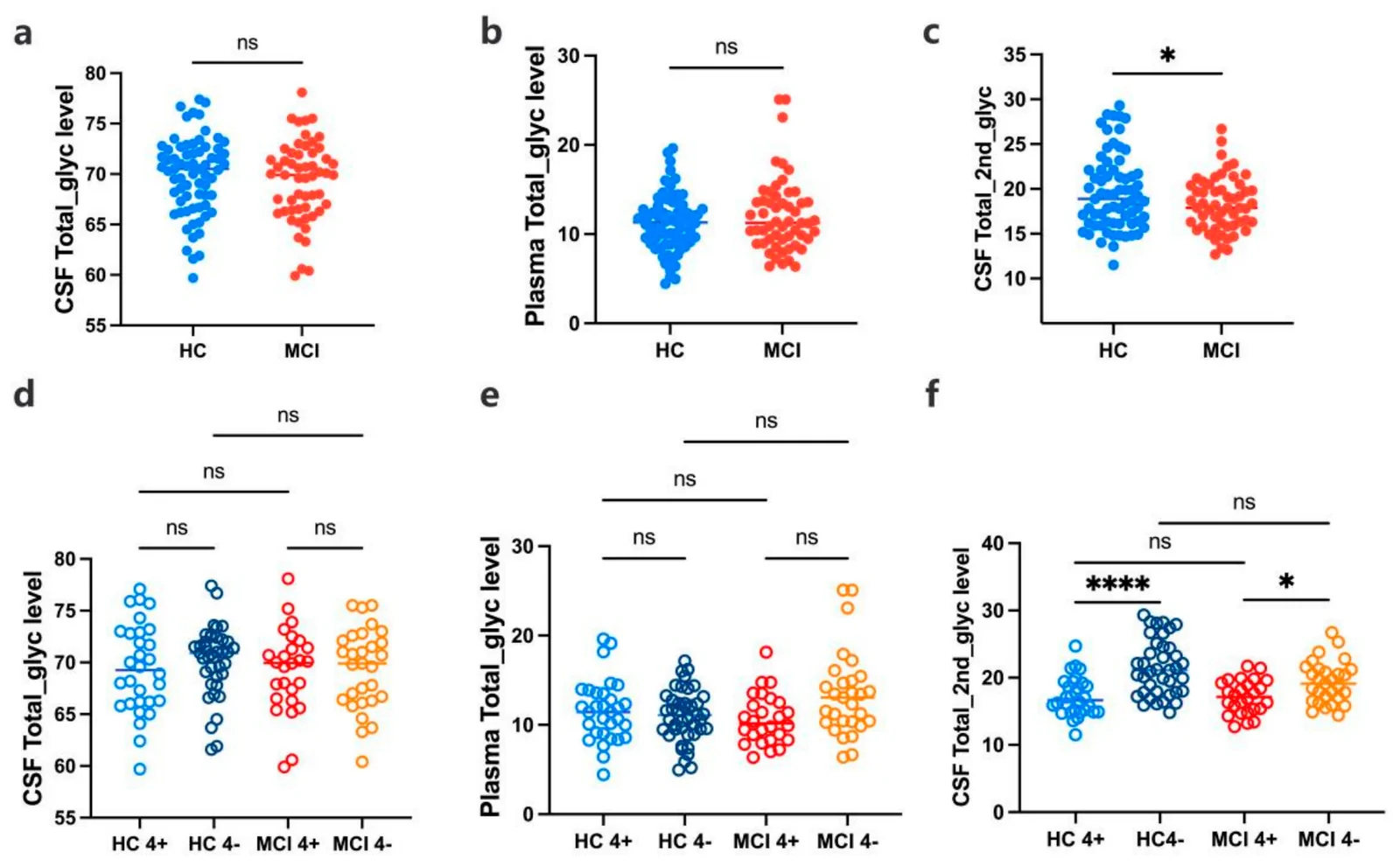
APOE glycosylation and secondary glycosylation levels in CSF and plasma of HC and MCI participants. (**a**,**b**) Total APOE glycosylation levels in CSF and plasma showed no significant differences between HC and MCI groups (HC: N = 75, MCI: N = 56). (**c**) Secondary APOE glycosylation in CSF was significantly reduced in MCI compared to HC participants (*p* = 0.041) (HC: N = 75, MCI: N = 56). (**d**) Within CSF, total glycosylation levels did not differ significantly by *APOE4* carrier status (HC 4+: N = 30, HC 4-: N = 45, MCI 4+: N = 26, MCI 4-: N = 30). (**e**) In plasma, total glycosylation levels did not differ significantly by *APOE4* carrier status (HC 4+: N = 30, HC 4-: N = 45, MCI 4+: N = 26, MCI 4-: N = 30). (**f**) Secondary glycosylation in CSF was significantly higher in non-*APOE4* carriers compared to non-carrier HCs (*p* < 0.0001). Among MCI participants, *APOE4* carriers exhibited significantly lower levels than *APOE4* non-carriers (*p* = 0.033). No significant differences were observed between other subgroups (HC 4+: N = 30, HC 4-: N = 45, MCI 4+: N = 26, MCI 4-: N = 30). *: *p* < 0.05, ****: *p* < 0.0001.

**Figure 2:**
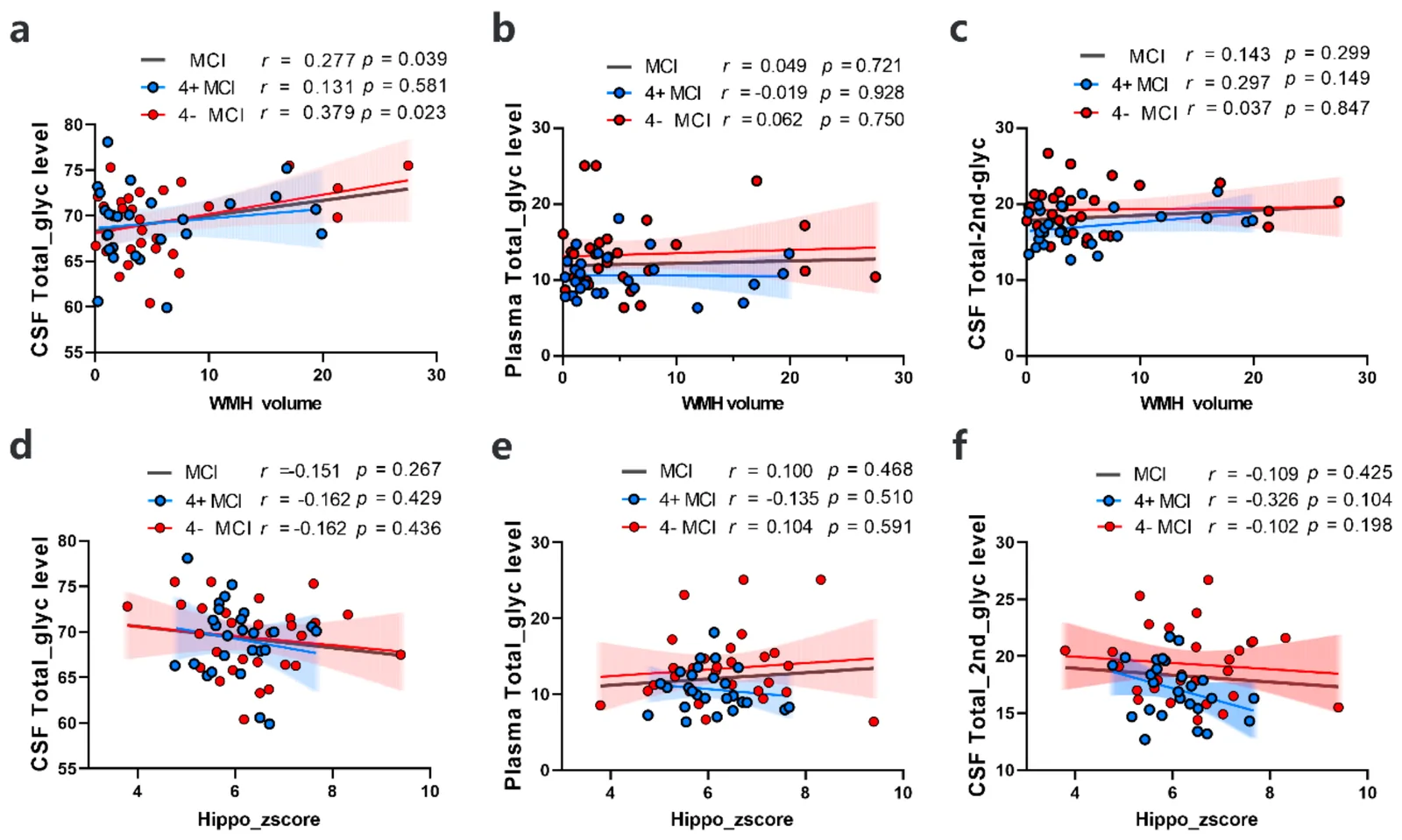
Correlations of APOE glycosylation with WMH volume and hippocampal volume in MCI participants. (**a**) CSF total glycosylation was positively correlated with WMH volume in MCI participants (*p* = 0.039), driven by significance in *APOE4* non-carriers (*p* = 0.023) (4+ MCI: N = 26; 4- MCI: N = 30). (**b**) Plasma total glycosylation showed a positive but non-significant association with WMH volume; subgroup trends varied by *APOE4* status (4+ MCI: N = 26; 4- MCI: N = 30). (**c**) CSF secondary glycosylation exhibited a non-significant negative correlation with WMH volume (4+ MCI: N = 26; 4- MCI: N = 30). (**d**) CSF total glycosylation showed a non-significant negative correlation with hippocampal volume in MCI participants, consistent across *APOE4* carriers (4+ MCI: N = 26; 4- MCI: N = 30). (**e**) Plasma total glycosylation demonstrated a non-significant positive correlation with hippocampal volume overall, with *APOE4* carriers showing a negative trend (4+ MCI: N = 26; 4- MCI: N = 30). (**f**) CSF secondary glycosylation displayed a non-significant negative association with hippocampal volume (4+ MCI: N = 26; 4- MCI: N = 30).

**Figure 3:**
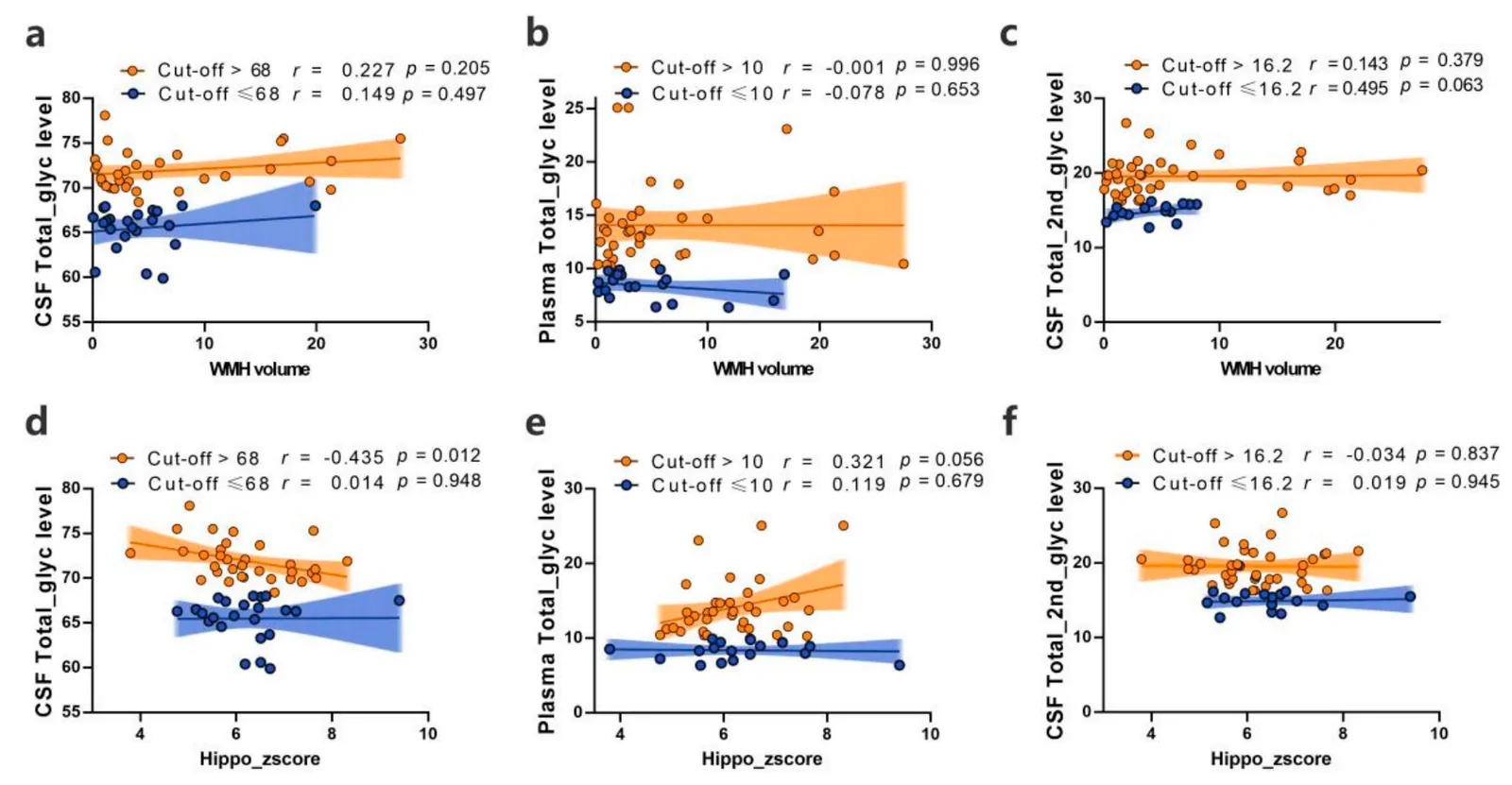
Associations between APOE glycosylation cut-off levels and neuroimaging markers in MCI participants. (**a**–**c**) No significant correlations were observed between WMH volume and CSF total glycosylation (cut-off 68), plasma total glycosylation (cut-off 10), or CSF secondary glycosylation (cut-off 16.2) (ns) ((**a**): >68: N = 33, ≤68: N = 23; (**b**): >10: N = 36, ≤10: N = 20; (**c**): >16.2: N = 40, ≤16.2: N = 16). (**d**) CSF total glycosylation levels of >68 were significantly negatively correlated with hippocampal volume (*p* = 0.012), while no correlation was observed in participants with levels of ≤68 (ns) (>68: N = 33, ≤68: N = 23). (**e**,**f**) Plasma total glycosylation (cut-off 10) and CSF secondary glycosylation (cut-off 16.2) showed no significant correlations with hippocampal volume (ns) ((**e**): >10: N = 36, ≤10: N = 20; (**f**): >16.2: N = 40, ≤16.2: N = 16).

**Figure 4:**
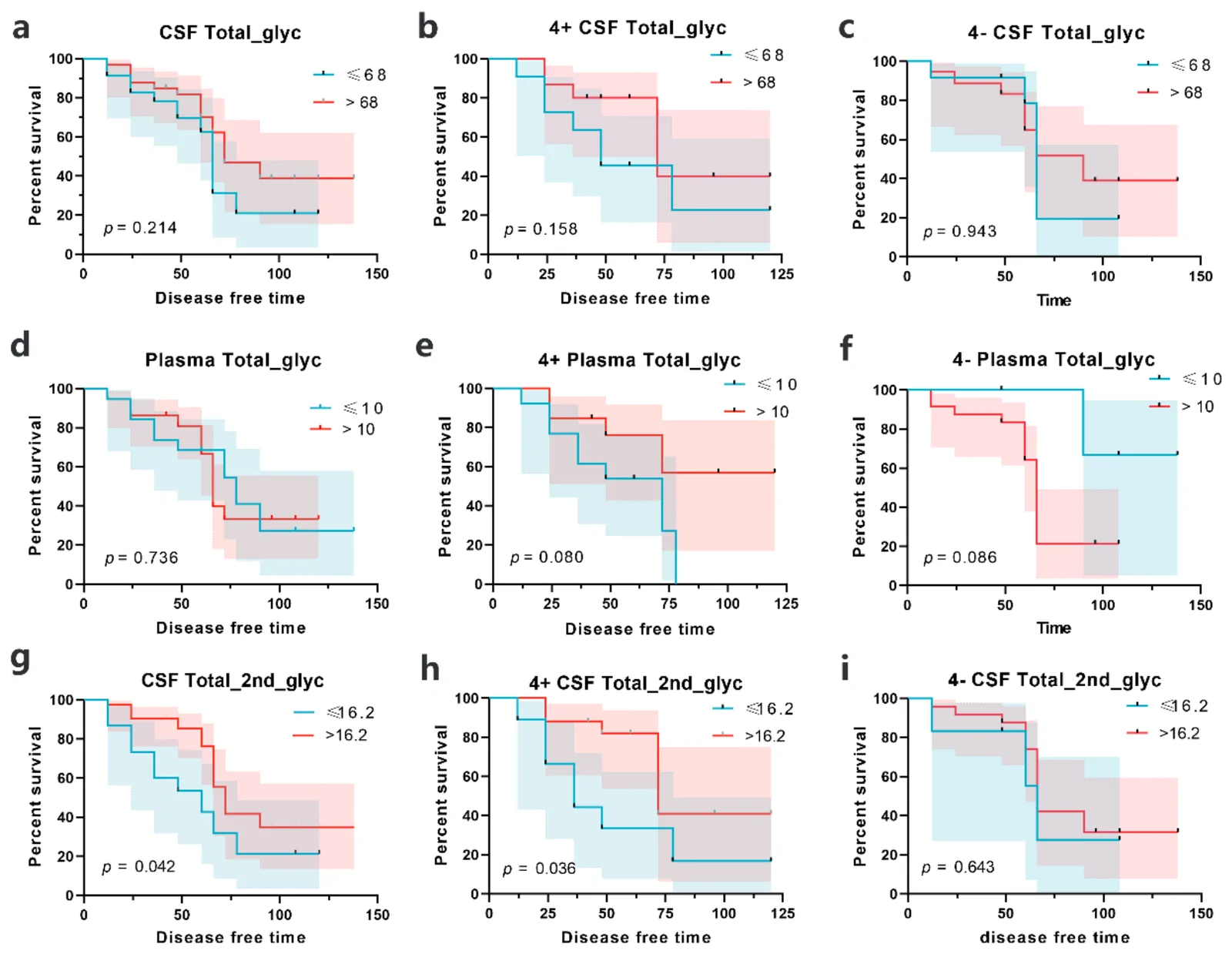
APOE glycosylation cut-off levels and risk of conversion from MCI to AD. (**a**–**c**) CSF total glycosylation (cut-off 68) showed no significant association with AD conversion time in all MCI participants or within *APOE4*+ and *APOE4*− subgroups (ns) ((**a**): >68: N = 33, ≤68: N = 23; (**b**): >68: N = 15, ≤68: N = 11; (**c**): >68: N = 18, ≤68: N = 12). (**d**–**f**) Plasma total glycosylation (cut-off 10) showed no significant differences in conversion time across MCI participants or *APOE4* subgroups (ns) ((**d**): >10: N = 36, ≤10: N = 20; (**e**): >10: N = 13, ≤10: N = 13; (**f**): >10: N = 23, ≤10: N = 7). (**g**) CSF secondary glycosylation (cut-off 16.2) was significantly associated with AD progression, with participants at or below the cut-off converting earlier than those above (*p* = 0.042) (>16.2: N = 40, ≤16.2: N = 16). (**h**) Within the *APOE4*+ subgroup, lower secondary glycosylation was also linked to earlier conversion (*p* = 0.036) (>16.2: N = 17, ≤16.2: N = 9). (**i**) No significant differences were observed in *APOE4*− participants (ns) (>16.2: N = 23, ≤16.2: N = 7).

**Figure 5:**
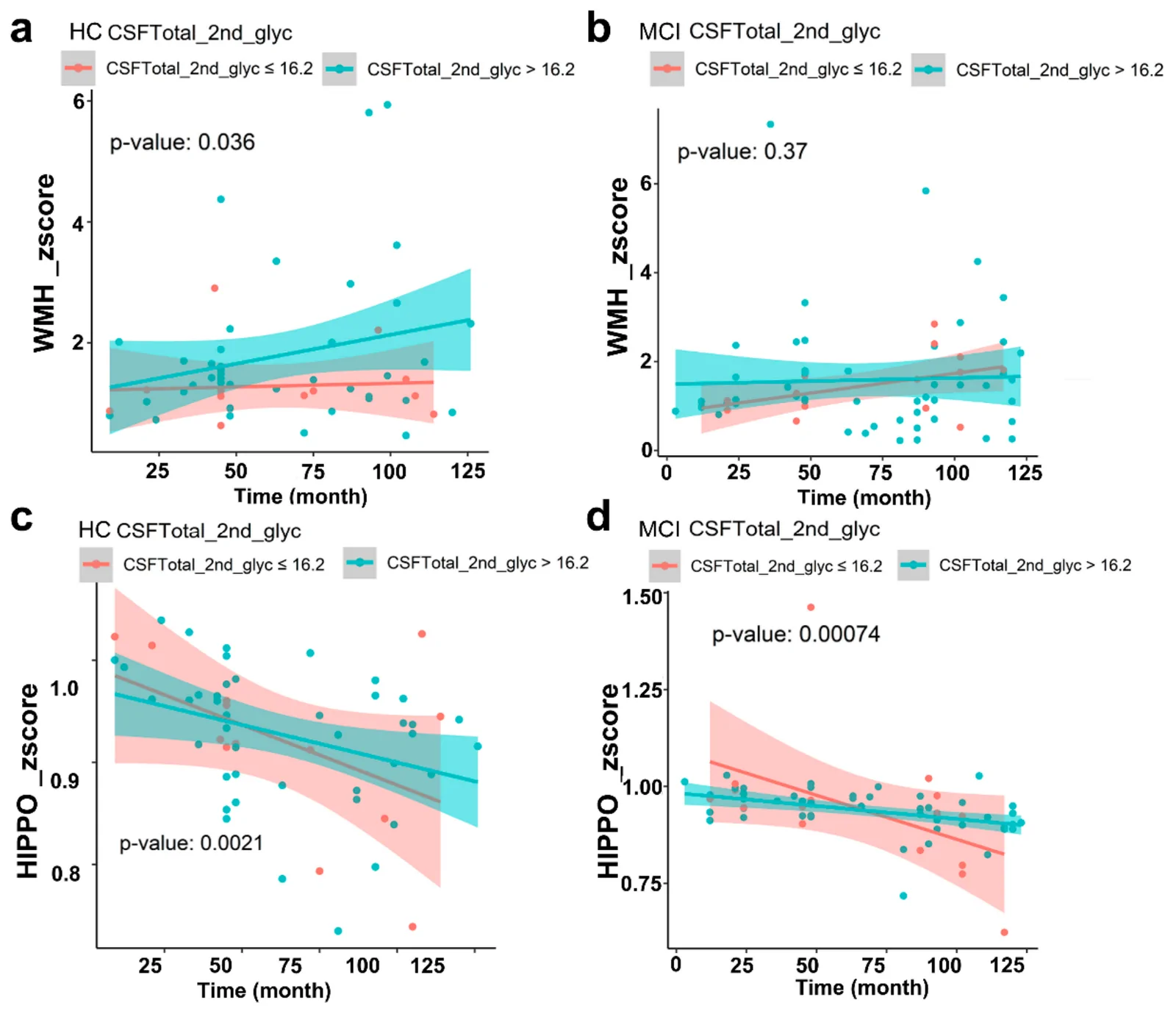
Predictive value of CSF secondary APOE glycosylation for structural brain changes. (**a**,**b**) While no significant differences were observed in the MCI group (ns), HC participants with CSF secondary glycosylation levels at or below the 16.2 cut-off exhibited significantly slower WMH progression compared to those above the cut-off. ((**a**): >16.2: N = 33, ≤16.2: N = 42; (**b**): >16.2: N = 40, ≤16.2: N = 16). (**c**,**d**) In both HC and MCI groups, hippocampal volume declined significantly faster in participants with CSF secondary glycosylation levels at or below the cut-off (≤16.2) compared to those with higher levels (*p* < 0.05). ((**c**): >16.2: N = 33, ≤16.2: N = 42; (**d**): >16.2: N = 40, ≤16.2: N = 16).

**Figure 6:**
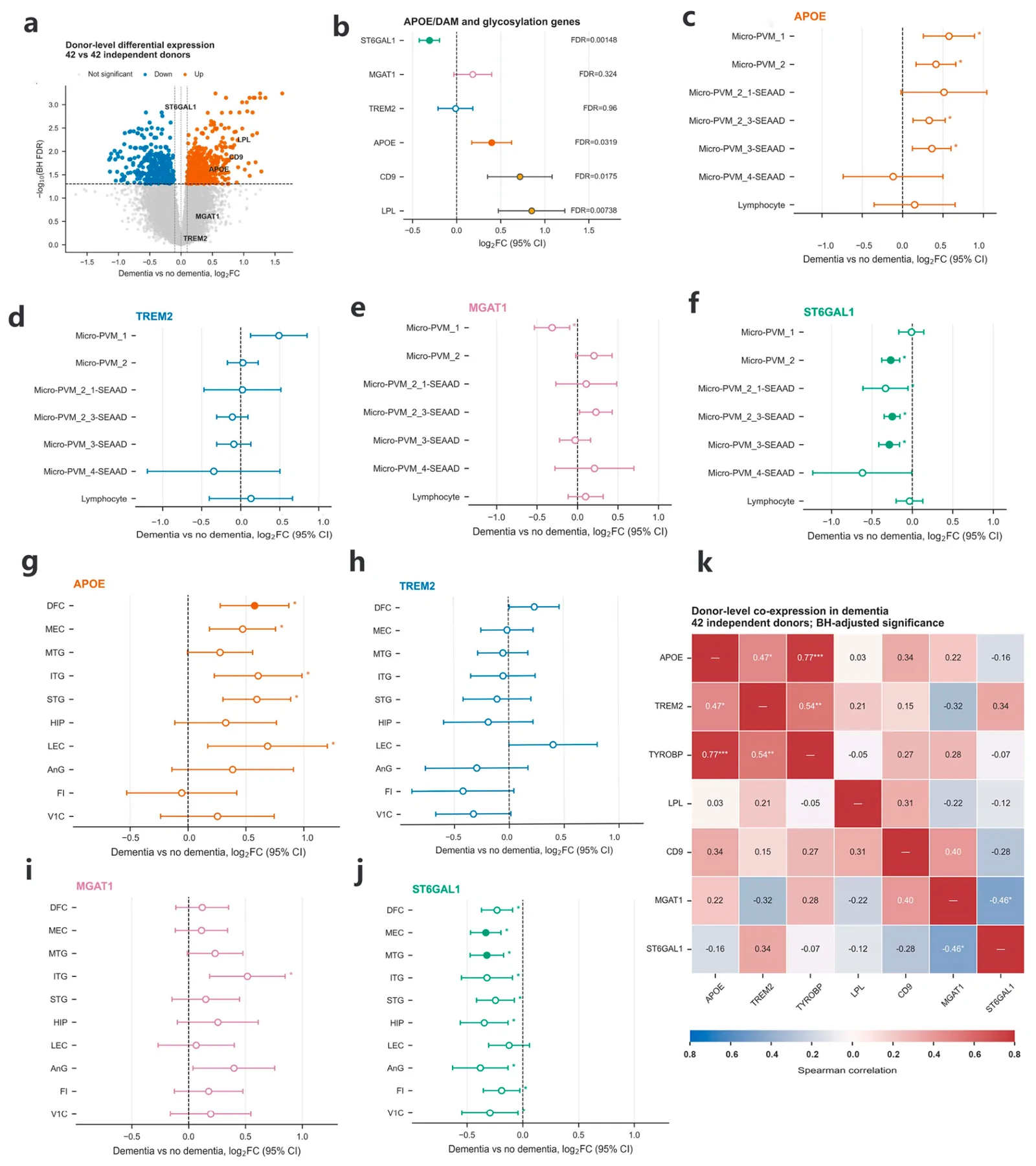
Global transcriptomic reprogramming and clinicopathological correlation of microglial glycosylation in dementia. (**a**) Overall donor-level pseudobulk differential expression of *APOE*/DAM and glycosylation-related genes in dementia. (**Up**) Volcano plot comparing dementia and no dementia groups using 42 independent donors per group. Orange and blue points denote genes with BH FDR < 0.05 that were up- and down-regulated, respectively; gray points were not significant. The horizontal dashed line indicates BH FDR = 0.05, the vertical dotted line indicates log2FC = 0, and the vertical dashed lines indicate |log2FC| = 0.1. (**Down**) Forest plot showing donor-level log2FC and approximate 95% confidence intervals for *ST6GAL1*, *MGAT1*, *TREM2*, *APOE*, *CD9*, and *LPL*. Filled markers indicate BH FDR < 0.05; open markers indicate non-significant results. (**b**–**f**) Dementia-versus-No dementia log2FC and approximate 95% confidence intervals for *APOE*, *TREM2*, *MGAT1*, and *ST6GAL1* across seven microglia-PVM supertypes. Filled markers indicate genome-wide BH FDR < 0.05 within the corresponding stratum. Asterisks indicate gene-wise BH FDR < 0.05 after correction of the prespecified gene across all 17 supertype and brain-region strata. The vertical dashed line indicates log2FC = 0. (**g**–**j**) Dementia-versus-No dementia log2FC and approximate 95% confidence intervals for *APOE*, *TREM2*, *MGAT1*, and *ST6GAL1* across ten SEA-AD brain regions. Filled markers indicate genome-wide BH FDR < 0.05 within the corresponding region. Asterisks indicate gene-wise BH FDR < 0.05 after correction of the prespecified gene across all 17 supertype and brain-region strata. The vertical dashed line indicates log2FC = 0. (**k**) Donor-level co-expression of *APOE*/DAM markers and glycosylation-related genes in dementia. Spearman correlations were calculated from donor pseudobulk logCPM values among the 42 independent dementia donors. Red and blue cells indicate positive and negative correlations, respectively, and each cell displays Spearman r. *p*-values were adjusted across the 21 unique gene pairs using the Benjamini–Hochberg procedure. Diagonal cells are marked with dashes. *: *p* < 0.05, **: *p* < 0.01, ***: *p* < 0.001.

**Table 1:** Demographic and clinical features of participants from the ADNI database at baseline.

Demographics	HC (n = 75)	MCI (n = 56)	*p* Value
Age (y)	72.5 ± 4.9	71.0 ± 7.3	0.150
Gender			0.818
Female	35	25	
Male	40	31	
Education duration (y)	16.2 ± 2.8	16.7 ± 2.5	0.337
MMSE	28.8 ± 1.4	28.1 ± 1.6	0.004
MoCA	25.8 ± 2.4	23.0 ± 3.4	*p* < 0.001
FAQ	0.1 ± 0.5	3.2 ± 4.3	*p* < 0.001
CSF Aβ_1–42_ (pg/mL)	984.6 ± 387.5	946.8 ± 383.1	0.684
CSF T-Tau (pg/mL)	246.4 ± 80.9	259.4 ± 119.6	0.987
CSF P-Tau (pg/mL)	22.7 ± 8.1	24.7 ± 12.7	0.923
*APOE4* carrier			0.377
(+)	30	26	
(-)	45	30	

Abbreviations: MMSE: Mini–Mental State Examination; FAQ: Functional Activities Questionnaire; HC: healthy control; MCI: mild cognitive impairment. Data are presented as mean ± standard deviation (SD).

## Data Availability

All data generated or analyzed during this study are included in this article and its Supplementary Material files. Further enquiries can be directed to the corresponding author.

## Supplementary Materials

The following supporting information can be downloaded at https://www.mdpi.com/article/10.3390/biomedicines14102206/s1. Table S1: The original *p*-values in each Figure and the corrected *p*-values after FDR adjustment.

## References

[1] Yamazaki Y., Zhao N., Caulfield T.R., Liu C.C., Bu G. (2019). Apolipoprotein e and alzheimer disease: Pathobiology and targeting strategies. Nat. Rev. Neurol..

[2] Serrano-Pozo A., Das S., Hyman B.T. (2021). Apoe and alzheimer’s disease: Advances in genetics, pathophysiology, and therapeutic approaches. Lancet Neurol..

[3] Lanfranco M.F., Sepulveda J., Kopetsky G., Rebeck G.W. (2021). Expression and secretion of apoe isoforms in astrocytes and microglia during inflammation. Glia.

[4] Moon H.J., Luo Y., Chugh D., Zhao L. (2024). Human apolipoprotein e glycosylation and sialylation: From structure to function. Front Mol. Neurosci..

[5] Flowers S.A., Rebeck G.W. (2020). Apoe in the normal brain. Neurobiol. Dis..

[6] Lawler P.E., Bollinger J.G., Schindler S.E., Hodge C.R., Iglesias N.J., Krishnan V., Coulton J.B., Li Y., Holtzman D.M., Bateman R.J. (2023). Apolipoprotein e o-glycosylation is associated with amyloid plaques and apoe genotype. Anal. Biochem.

[7] Nedelkov D., Tsokolas Z.E., Rodrigues M.S., Sible I., Han S.D., Kerman B.E., Renteln M., Mack W.J., Pascoal T.A., Yassine H.N. (2025). Increased cerebrospinal fluid and plasma apoe glycosylation is associated with reduced levels of alzheimer’s disease biomarkers. Alzheimers Res. Ther..

[8] Meuret C.J., Hu Y., Smadi S., Bantugan M.A., Xian H., Martinez A.E., Krauss R.M., Ma Q.L., Nedelkov D., Yassine H.N. (2023). An association of csf apolipoprotein e glycosylation and amyloid-beta 42 in individuals who carry the apoe4 allele. Alzheimers Res. Ther..

[9] Chua C.C., Lim M.L., Wong B.S. (2010). Altered apolipoprotein e glycosylation is associated with abeta(42) accumulation in an animal model of niemann-pick type c disease. J. Neurochem..

[10] Hu Y., Meuret C., Go S., Yassine H.N., Nedelkov D. (2020). Simple and fast assay for apolipoprotein e phenotyping and glycotyping: Discovering isoform-specific glycosylation in plasma and cerebrospinal fluid. J. Alzheimers Dis..

[11] Zhang H., Wu L.M., Wu J. (2011). Cross-talk between apolipoprotein e and cytokines. Mediat. Inflamm..

[12] Zhao A., Jiao Y., Ye G., Kang W., Tan L., Li Y., Deng Y., Liu J. (2022). Soluble trem2 levels associate with conversion from mild cognitive impairment to alzheimer’s disease. J. Clin. Investig..

[13] Petersen R.C., Smith G.E., Waring S.C., Ivnik R.J., Tangalos E.G., Kokmen E. (1999). Mild cognitive impairment: Clinical characterization and outcome. Arch. Neurol..

[14] Ding Y., Wang L., Liu J., Deng Y., Jiao Y., Zhao A. (2025). Distinct csf α-synuclein aggregation profiles associated with alzheimer’s disease phenotypes and mci-to-ad conversion. J. Prev. Alzheimers Dis..

[15] Jack C.R., Marjanska M., Wengenack T.M., Reyes D.A., Curran G.L., Lin J., Preboske G.M., Poduslo J.F., Garwood M. (2007). Magnetic resonance imaging of alzheimer’s pathology in the brains of living transgenic mice: A new tool in alzheimer’s disease research. Neuroscientist.

[16] DeCarli C., Rajan K.B., Jin L.W., Hinman J., Johnson D.K., Harvey D., Fornage M. (2025). Wmh contributions to cognitive impairment: Rationale and design of the diverse vcid study. Stroke.

[17] Bettio L.E.B., Rajendran L., Gil-Mohapel J. (2017). The effects of aging in the hippocampus and cognitive decline. Neurosci. Biobehav. Rev..

[18] Kockx M., Traini M., Kritharides L. (2018). Cell-specific production, secretion, and function of apolipoprotein e. J. Mol. Med..

[19] Miranda A.M., Ashok A., Chan R.B., Zhou B., Xu Y., McIntire L.B., Area-Gomez E., Di Paolo G., Duff K.E., Oliveira T.G. (2022). Effects of apoe4 allelic dosage on lipidomic signatures in the entorhinal cortex of aged mice. Transl. Psychiatry.

[20] Christie R.H., Chung H., Rebeck G.W., Strickland D., Hyman B.T. (1996). Expression of the very low-density lipoprotein receptor (vldl-r), an apolipoprotein-e receptor, in the central nervous system and in alzheimer’s disease. J. Neuropathol. Exp. Neurol..

[21] Safina D., Schlitt F., Romeo R., Pflanzner T., Pietrzik C.U., Narayanaswami V., Edenhofer F., Faissner A. (2016). Low-density lipoprotein receptor-related protein 1 is a novel modulator of radial glia stem cell proliferation, survival, and differentiation. Glia.

[22] Ke L.Y., Chan H.C., Chen C.C., Chang C.F., Lu P.L., Chu C.S., Lai W.T., Shin S.J., Liu F.T., Chen C.H. (2020). Increased apoe glycosylation plays a key role in the atherogenicity of l5 low-density lipoprotein. Faseb J..

[23] Sheng H., Laskowitz D.T., Bennett E., Schmechel D.E., Bart R.D., Saunders A.M., Pearlstein R.D., Roses A.D., Warner D.S. (1998). Apolipoprotein e isoform-specific differences in outcome from focal ischemia in transgenic mice. J. Cereb. Blood Flow Metab..

[24] Mirza S.S., Saeed U., Knight J., Ramirez J., Stuss D.T., Keith J., Nestor S.M., Yu D., Swardfager W., Rogaeva E. (2019). Apoe ε4, white matter hyperintensities, and cognition in alzheimer and lewy body dementia. Neurology.

[25] Raber J., Silbert L.C. (2023). Role of white matter hyperintensity in effects of apolipoprotein e on cognitive injury. Front. Hum. Neurosci..

[26] Kern K.C., Zagzoug M.S., Gottesman R.F., Wright C.B., Leigh R. (2024). Blood-brain barrier disruption and increased free water are associated with worse cognitive performance in patients with chronic cerebrovascular disease. Neuroimage Clin..

[27] Zeng Q., Li K., Luo X., Wang S., Xu X., Jiaerken Y., Liu X., Hong L., Hong H., Li Z. (2022). The association of enlarged perivascular space with microglia-related inflammation and alzheimer’s pathology in cognitively normal elderly. Neurobiol. Dis..

[28] Hase Y., Horsburgh K., Ihara M., Kalaria R.N. (2018). White matter degeneration in vascular and other ageing-related dementias. J. Neurochem..

[29] Kaskikallio A., Karrasch M., Koikkalainen J., Lötjönen J., Rinne J.O., Tuokkola T., Parkkola R., Grönholm-Nyman P. (2021). Effects of white matter hyperintensities on verbal fluency in healthy older adults and mci/ad. Front. Aging Neurosci..

[30] Zhang J., Xie L., Cheng C., Liu Y., Zhang X., Wang H., Hu J., Yu H., Xu J. (2023). Hippocampal subfield volumes in mild cognitive impairment and alzheimer’s disease: A systematic review and meta-analysis. Brain Imaging Behav..

[31] Liu S., Breitbart A., Sun Y., Mehta P.D., Boutajangout A., Scholtzova H., Wisniewski T. (2014). Blocking the apolipoprotein e/amyloid β interaction in triple transgenic mice ameliorates alzheimer’s disease related amyloid β and tau pathology. J. Neurochem..

[32] Gabitto M.I., Travaglini K.J., Rachleff V.M., Kaplan E.S., Long B., Ariza J., Ding Y., Mahoney J.T., Dee N., Goldy J. (2024). Integrated multimodal cell atlas of alzheimer’s disease. Nat. Neurosci..

[33] Krasemann S., Madore C., Cialic R., Baufeld C., Calcagno N., El Fatimy R., Beckers L., O’Loughlin E., Xu Y., Fanek Z. (2017). The trem2-apoe pathway drives the transcriptional phenotype of dysfunctional microglia in neurodegenerative diseases. Immunity.

[34] Giusti V., Park J., Giusto E., Masatti L., Ramos-Gonzalez P., Iovino L., Sandre M., Emmi A., Kaur G., Coletto E. (2026). Atypical chemokine receptor 3 regulates synaptic removal in disease astrocytes. Mol. Neurodegener..

