# Supplementary material for: CSF APOE Secondary Glycosylation Predicts Cognitive Decline in Mild Cognitive Impairment
Source: Biomedicines. 2026 Sep 29;14(10):2206. doi: 10.3390/biomedicines14102206 (PMC13701876; doi:10.3390/biomedicines14102206)
Supplement: Supplementary file 1 [file biomedicines-14-02206-s001.zip › biomedicines-4529045-supplementary.pdf]

Supplement Table S1: The original P-values in each Figure and the corrected P-values after FDR adjustment.

Figure	original P-values	corrected P-values
1a	0.4515	0.8620
1b	0.1929	0.4101
1c	0.0483	0.1691
1d	0.8582	1.0000
	0.9976	1.0000
	1.0000	1.0000
	0.7728	1.0000
	0.8503	1.0000
	0.9984	1.0000
1e	0.9044	1.0000
	0.7498	1.0000
	0.1953	0.4101
	0.9741	1.0000
	0.0288	0.1512
	0.0231	0.1512
1f	0.0001	0.0011
	0.9999	1.0000
	0.0701	0.1950
	0.0001	0.0011
	0.0400	0.1680
	0.0743	0.1950
2a	0.0390	0.3510
	0.5810	0.7599
	0.0230	0.3510
2b	0.7210	0.8438
	0.9280	0.9280
	0.7500	0.8438
2c	0.2990	0.7599
	0.1490	0.6705
	0.8470	0.8968
2d	0.2670	0.7599
	0.4290	0.7599
	0.4360	0.7599
2e	0.4680	0.7599
	0.5100	0.7599
	0.5910	0.7599
2f	0.4250	0.7599
	0.1040	0.6240
	0.1980	0.7128
3a	0.2050	0.6150
	0.4970	0.9940
3b	0.9960	0.9960
	0.6530	0.9960
3c	0.3790	0.9096
	0.0630	0.2520

3d	0.0120	0.1440
	0.9480	0.9960
3e	0.0560	0.2520
	0.6790	0.9960
3f	0.8370	0.9960
	0.9450	0.9960
4a	0.2140	0.3210
4b	0.1580	0.2844
4c	0.9430	0.9430
4d	0.7360	0.8280
4e	0.0800	0.1935
4f	0.0860	0.1935
4g	0.0420	0.1890
4h	0.0360	0.1890
4i	0.6430	0.8267
5a	0.0360	0.0480
5b	0.3700	0.3700
5c	0.0021	0.0042
5d	0.0007	0.0030
